# Development and application of a PCR–RFLP assay revealing widespread distribution of the pyrethroid resistance-associated VGSC V1016G mutation in *Aedes albopictus* from Guangyuan City, Sichuan Province of China

**DOI:** 10.1186/s13071-025-07116-z

**Published:** 2025-11-24

**Authors:** Xiaoqiang Lu, Qiongyao Zhao, Binyu Yang, Qiangan Zhang, Jie He, Zeying Zhou, Tu Yan, Yongchao Jia, Xinghui Qiu

**Affiliations:** 1 Guangyuan Center for Disease Control and Prevention, No. 203 Binhe North Rode, Dongba District, Guangyuan, 628040 Sichuan China; 2 State Key Laboratory of Animal Biodiversity Conservation and Integrated Pest Management, Institute of Zoology, Chinese Academy of Sciences, 1-5 Beichen West Road, Chaoyang District, Beijing, 100101 China

**Keywords:** *Aedes albopictus*, Voltage-gated sodium channel, Insecticide resistance, V1016G mutation, Pyrethroid insecticides, Polymerase chain reaction–restriction fragment length polymorphism (PCR–RFLP)

## Abstract

**Background:**

*Aedes albopictus* is a primary vector of multiple arboviruses, including dengue, chikungunya, yellow fever, and Zika virus. Its control relies heavily on pyrethroid insecticides. The V1016G mutation in the voltage-gated sodium channel (VGSC) is a well-documented mechanism conferring pyrethroid resistance in *Ae. albopictus*, which directly challenges the efficacy of pyrethroid-based control. Understanding of the status of insecticide resistance will offer insights to inform evidence-based vector management. However, current phenotypic monitoring is laborious and time-consuming, highlighting the need for rapid and reliable genotyping tools.

**Methods:**

To detect the V1016G mutation, we developed a polymerase chain reaction–restriction fragment length polymorphism (PCR–RFLP) assay. This assay was then applied to genotype 208 field-collected *Ae. albopictus* mosquitoes. These samples were collected in 2024 from seven counties/districts within Guangyuan City, a prefecture in northern Sichuan, China.

**Results:**

The PCR–RFLP assay demonstrated 100% concordance with Sanger sequencing results. Genotyping confirmed the widespread presence of the 1016G allele, with frequencies ranging from 3.13% to 14.06%. The resistance allele (1016G) was exclusively detected in heterozygotes, and all populations conformed to Hardy–Weinberg equilibrium (*P* > 0.05). Furthermore, no significant temporal changes in allele frequencies were detected between 2020 and 2024 across the populations (*P* > 0.05).

**Conclusions:**

This study established a cost-effective and reliable PCR–RFLP assay for detecting the V1016G mutation in *Ae. albopictus*, and demonstrated the widespread distribution of this mutation across Guangyuan City, Sichuan Province of China.

**Graphical abstract:**

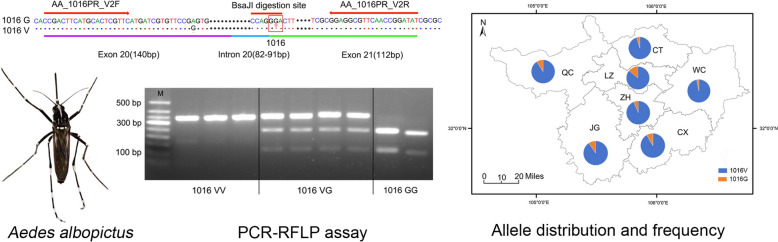

**Supplementary Information:**

The online version contains supplementary material available at 10.1186/s13071-025-07116-z.

## Background

*Aedes albopictus* (Skuse, 1894) is a primary vector for multiple arboviruses, including dengue virus (DENV), chikungunya virus, yellow fever virus, and Zika virus [1]. DENV alone causes approximately 100 million global cases annually [2, 3], with incidence potentially rising due to expanding global trade and tourism. From 2005 to 2020, 94,478 dengue cases were reported across 30 provincial-level administrative divisions in mainland China [4]. Originally native to Southeast Asia, the western Pacific, and islands in the Indian Ocean, this mosquito species has now spread to all continents except Antarctica [2, 5]. In the absence of effective therapeutics or accessible vaccines, insecticide-based vector population control remains critical for mitigating disease transmission. However, the continuous use of insecticides has led to the development of insecticide resistance in *Ae. albopictus* populations [6–10]. The known resistance mechanisms in *Ae. albopictus* primarily include target-site resistance and metabolic resistance. Target-site resistance is well established; for instance, specific mutations in the voltage-gated sodium channel (VGSC) can confer pyrethroid resistance by reducing channel sensitivity [6]. Metabolic resistance, on the other hand, is known to be mediated by various detoxification enzymes, including cytochrome P450, glutathione S-transferase, and carboxylesterase [11].

The VGSC, encoded by the *VGSC* gene, serves as the primary target site of pyrethroid insecticides. In *Ae. albopictus*, three resistance-associated mutations in this channel have been identified, i.e. V1016G, I1532T, and F1534C/L/S [10–17]. Previous studies have demonstrated that homozygous *Ae. albopictus* strains carrying the 1016GG, 1534SS, and 1534CC genotypes exhibit resistance ratios of 25–32, 4–5.7, and 2.4–4.3, respectively, against pyrethroids such as permethrin, etofenprox, and deltamethrin [6]. Although the I1532T mutation has been detected in numerous resistant populations, its functional role in insecticide resistance remains unclear. To date, the V1016G mutation is considered the most well-documented key mutation conferring pyrethroid resistance.

Monitoring insecticide resistance is essential for effective vector control. Resistance assessment generally includes both phenotypic and genotypic approaches, with combined data providing the most comprehensive picture. Currently, insecticide resistance surveillance in China relies predominantly on larval and adult mosquito bioassays [7, 8]. While these bioassays provide crucial phenotypic data, they are often labor-intensive and time-consuming, which limits their suitability for rapid, large-scale resistance surveillance. Advances in elucidating the molecular mechanisms have enabled genotypic monitoring. In fact, several molecular diagnostic platforms have been developed as alternatives or complementary approaches. Specifically, the reported genotyping methods for resistance-associated sodium channel mutations in *Ae. albopictus* include direct DNA sequencing [16], allele-specific PCR (AS–PCR) [3, 18], and PCR coupled with mass spectrometry (PCR–MS) [19]. The polymerase chain reaction–restriction fragment length polymorphism (PCR–RFLP) assay, which integrates PCR with enzyme digestion, is often used to detect genetic variation based on DNA fragment length polymorphism [20–22]. However, a validated PCR–RFLP assay specifically for the V1016G mutation in *Ae. albopictus* was not available prior to this study.

The objectives of this study were twofold: (1) to develop and validate a PCR–RFLP assay for detecting the V1016G mutation in *Ae. albopictus*, and (2) to apply this assay to assess the current frequency and distribution of V1016G in field populations across Guangyuan City. Guangyuan City is located in northern Sichuan Province of China, covering a total area of 16,319 km^2^, with a population of nearly 3 million. *Aedes albopictus*, a dominant mosquito species in this region [23], poses a significant concern, as imported dengue fever cases have been reported there over the past decade [10].

## Methods

### *Aedes albopictus* samples

**Fig. 1:**
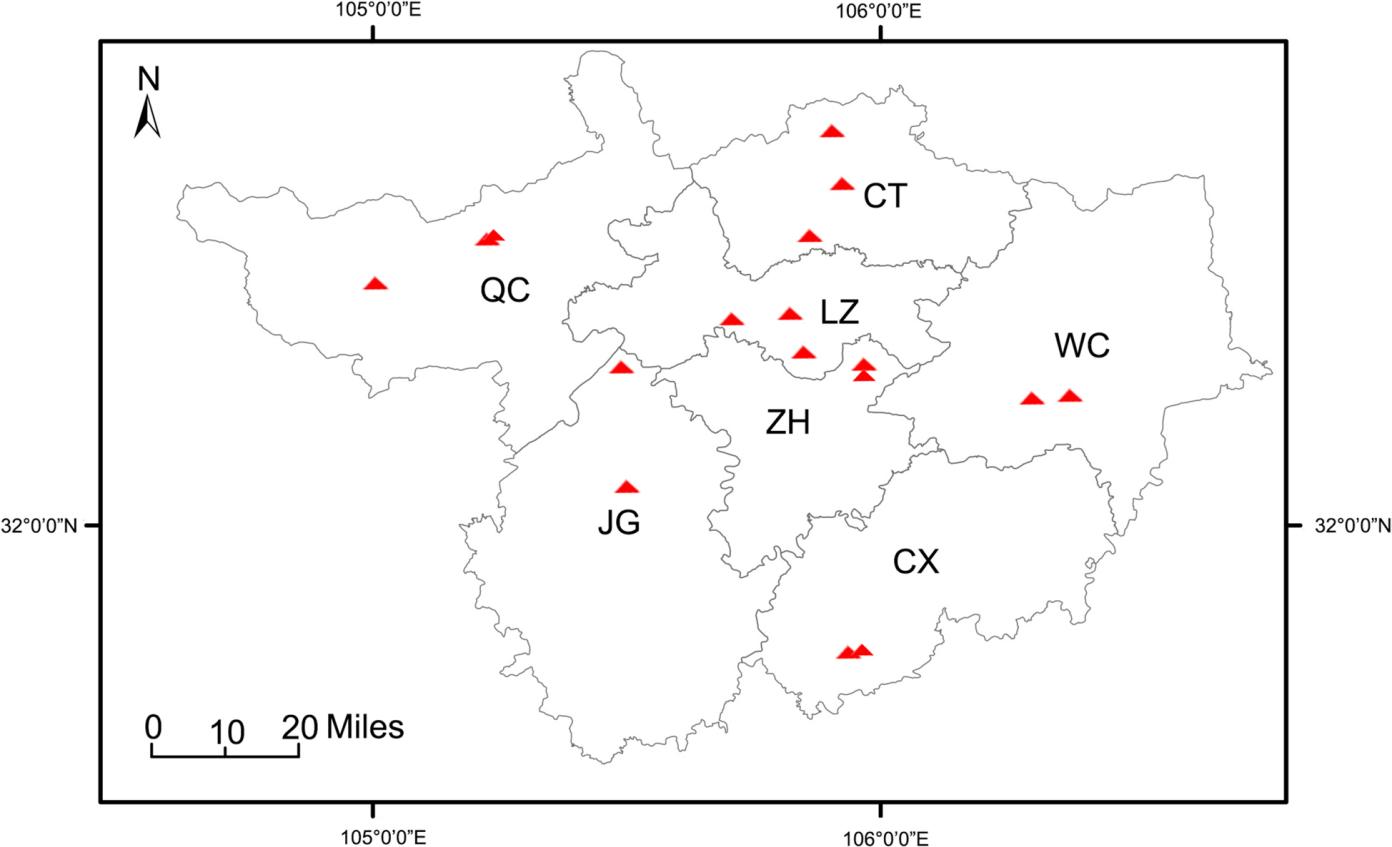
Geographical locations of the sampling sites. CT = Chao-Tian District; CX = Cang-Xi County; JG = Jian-Ge County; LZ = Li-Zhou District; QC = Qing-Chuan County, WC = Wang-Cang County; ZH = Zhao-Hua District. Red triangles indicate sampling sites

Adult *Ae. albopictus* mosquitoes were collected using the human landing catch method from seven counties/districts in Guangyuan City between June and July 2024 (Fig. 1). After collection, the specimens were killed by freezing, morphologically identified, and individually preserved in microcentrifuge tubes containing absolute ethanol at −80 °C.

### Amplification of the *VGSC* gene fragment containing the 1016 locus

**Fig. 2:**
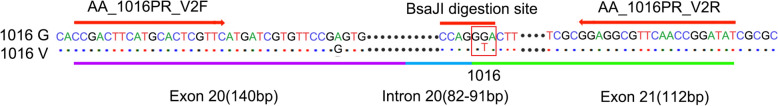
Partial DNA sequence of domain II of the *Ae. albopictus* *VGSC* gene. Red arrows indicate the forward and reverse primer-corresponding region. The BsaJI restriction site, and partial region of exon/intron are presented

Genomic DNA was extracted using the MagaBio Insect Genomic DNA Purification Kit (BSC33S1E) based on the magnetic bead method, and stored at −20 °C for subsequent use. Primers were designed based on consensus sequences derived from *VGSC* gene sequences obtained from 255 *Ae. albopictus* individuals previously collected across Guangyuan City [10], and their specificity was confirmed by the Basic Local Alignment Search Tool (BLAST) search results against the National Center for Biotechnology Information (NCBI) database (www.ncbi.nlm.nih.gov). The forward primer AA_1016PR_V2F (5′-CCGACTTCATGCACTCGTTC-3′) and the reverse primer AA_1016PR_V2R (5′-ATATCCGGTTGAACGCCTCC-3′) were used to amplify an approximately 350-base-pair (bp) DNA fragment spanning domain II of the *VGSC* containing codon 1016 (Fig. 2).

PCR amplification was performed in a 25 μl reaction mixture containing 12.5 μl of 2× Taq Master Mix (TSE102, Tsingke Biotechnology, China), 1 μl of each primer (10 μM), 1 μl of DNA, and 9.5 μl of double-distilled water (ddH_2_O). The reaction procedure was as follows: initial denaturation at 98 °C for 10 s, followed by 37 cycles of 98 °C for 10 s, 56 °C for 12 s, and 72 °C for 8 s, and a final extension at 72 °C for 3 min. The PCR products were subsequently subjected to restriction enzyme digestion analysis.

### Establishment of a PCR–RFLP assay for genotyping *VGSC*- V1016G

Restriction site analysis using SnapGene 5.2 software identified BsaJI as a suitable restriction endonuclease for discriminating the resistance allele (1016G). BsaJI recognizes the restriction site 5′…C^▼^CNNGG…3′. Digestion of the PCR product with BsaJI yielded fragments of approximately 115 bp and 240 bp for the 1016G allele (Fig. 2).

The restriction digestion reaction was performed in a 20 μl volume, consisting of 2 μl of 10× rCutSmart™ Buffer (B6004, New England Biolabs), 5 μl of the *VGSC* 1016 PCR amplicon, 1 μl of BsaJI (10 U/μl) (R0536, New England Biolabs), and 12 μl of ddH_2_O. The mixture was incubated at 60 °C for 2 h. The enzyme-digested products were separated by electrophoresis on a 2% agarose gel and visualized under ultraviolet (UV) light. Individual genotypes were determined based on the observed DNA banding patterns.

### Hardy–Weinberg equilibrium test and statistical analyses

Hardy–Weinberg equilibrium (HWE) was assessed using the exact test with default Markov chain parameters in the web-based version of Genepop 4.7 [24, 25]. Allele frequencies of the *VGSC*-1016 in Guangyuan mosquito populations collected in 2020 [10] and in 2024 (this study) were compared using the Chi-square or Fisher's exact test in SPSS 25 software.

## Results

### A PCR–RFLP assay to genotype the V1016G mutation

Gel electrophoresis of the PCR products showed a single band of approximately 350 bp, consistent with the expected amplicon size using the specific primer sets (Fig. S1). The presence of a BsaJI restriction site in the resistance allele (1016G) enables its PCR product to be cleaved into fragments of 115 bp and ~240 bp. As illustrated in Fig. S1, the expected band patterns after BsaJI digestion were as follows: two bands (115 bp and ~240 bp) for resistant homozygotes (1016 GG), three bands (115 bp, ~240 bp, and ~350 bp) for heterozygotes (1016 VG), and a single undigested band (~ 350 bp) for wild-type homozygotes (1016 VV).

To validate the accuracy of the PCR–RFLP assay, 48 randomly selected samples were subjected to Sanger sequencing of the PCR amplicons. The genotyping results obtained by PCR–RFLP showed 100% concordance with those from Sanger sequencing, covering wild-type homozygotes, resistant heterozygous, and resistant homozygous genotypes (Table 1).

**Table 1:** Comparison of genotyping results obtained by Sanger sequencing and by the PCR–RFLP assay

		PCR–RFLP			
		1016 VV	1016 VG	1016 GG	Total
Sequencing	1016 VV	39	0	0	39
	1016 VG	0	7	0	7
	1016 GG	0	0	2	2
	In total	39	7	2	48

### Distribution frequency of the V1016G mutation in field populations of *Ae. albopictus* from Guangyuan City

Genotyping of the V1016G mutation was performed on 208 field-collected *Ae. albopictus* samples from seven counties/districts of Guangyuan City using the newly established PCR–RFLP assay. The results showed that the 1016G allele was widely distributed in Guanyuan City. Notably, this resistance allele was exclusively found in heterozygous individuals (VG genotype), with heterozygote frequencies ranging from 6.25% to 28.13% (Table 2). All seven sampled populations conformed to HWE expectations for the genotypes at the locus carrying the V1016G mutation (*P* > 0.05; Table 2). Notably, no GG homozygotes were detected despite an expected frequency range of 0.1–2.0% based on allele frequencies, which is consistent with stochastic expectations given the sample sizes.

**Table 2:** Individual genotype frequencies of *VGSC* at position 1016 in field populations of *Ae. albopictus* in Guangyuan City, Sichuan Province of China, in 2024

Population	n	1016			HWE test
		VV	VG	GG	*P*-value
CT	32	29 (90.63%)	3 (9.38%)	0	1.0000
CX	36	30 (83.33%)	6 (16.67%)	0	1.0000
JG	32	26 (81.25%)	6 (18.75%)	0	1.0000
LZ	32	23 (71.88%)	9 (28.13%)	0	1.0000
QC	28	23 (82.14%)	5 (17.86%)	0	1.0000
WC	32	30 ( 93.75%)	2 (6.25%)	0	1.0000
ZH	16	14 (87.50%)	2 (12.50%)	0	1.0000
In total	208	175 (84.13%)	33 (15.87%)	0	–

HWE tests were run in the web-based version of Genepop 4.7 using exact tests with default Markov chain parameters. The *P*-values are given by the software. *P*-values > 0.05 indicate conformity to HWE

The frequencies of the resistance allele (1016G) ranged from 3.13% (in Wang-Cang County) to 14.06% (in Li-Zhou District) in 2024, with no significant differences observed among the counties/districts (*P* = 0.356, Table 3). Furthermore, comparison with 2020 data [10] revealed no statistically significant temporal changes in allele frequencies across the populations (*P* > 0.05) (Table 3).

**Table 3:** The frequencies of *VGSC* alleles in seven *Ae. albopictus* populations collected from Guangyuan City, Sichuan Province of China in 2024 and 2020

Population	Year	n	1016 V	1016G	Test	p-value
CT	2024	32	95.31	4.69	Fisher	0.334
	2020	36	90.28	9.72		
CX	2024	36	91.67	8.33	Fisher	0.494
	2020	35	95.71	4.29		
JG	2024	32	90.63	9.38	Chi-square	0.831
	2020	36	91.67	8.33		
LZ	2024	32	85.94	14.06	Chi-square	0.612
	2020	40	88.75	11.25		
QC	2024	28	91.07	8.93	Fisher	0.086
	2020	36	98.61	1.39		
WC	2024	32	96.88	3.13	Fisher	0.684
	2020	36	94.44	5.56		
ZH	2024	16	93.75	6.25	Fisher	0.217
	2020	36	83.33	16.67		

Data for year 2020 were reported in Zhao et al. [10]

## Discussion

Although DNA sequencing remains the gold standard for detecting genetic mutations, its reliance on costly instrumentation and specialized expertise limits its accessibility for routine resistance monitoring in grassroots institutions. As existing alternatives, AS–PCR [3, 18], while user-friendly, suffers from elevated false-positive rates due to primer mismatch risk [3], whereas PCR–MS [19] enables multiplex detection, but requires costly MS platforms.

To address the need for a simple, reliable, and cost-effective genotyping tool, in this study we developed and validated a PCR–RFLP assay suitable for genotyping the key V1016G mutation in *Ae. albopictus*. The primers used were optimally designed based on sequencing data of the *VGSC* gene from local *Ae. albopictus* samples [10], and further verified through BLAST analysis against the NCBI database. Using this pair of primers, a gene-specific fragment of ~350 bp covering the target position 1016 of the *VGSC* gene was consistently amplified (Fig. 2). Digestion with the allele-specific restriction enzyme (BsaJI) enabled accurate genotyping of the 1016 locus, achieving 100% concordance with Sanger sequencing results (Table 1).

We selected the PCR–RFLP approach for its recognized advantages in simplicity, cost-effectiveness, and robustness. However, in comparison to the existing AS–PCR method [3], our assay is more time-consuming and costly, primarily due to the additional expense of restriction enzymes and the extra step of enzymatic digestion. Another limitation of this study lies in the relatively narrow geographical scope of the sampled mosquitoes, which may not capture the full spectrum of genetic diversity. Consequently, the 100% accuracy reported for our PCR–RFLP assay might be overestimated, as it could be affected by unknown polymorphisms within the amplified region that were not present in our restricted sample set.

The availability of the PCR–RFLP tool makes it possible to extensively investigate the current occurrence of the insecticide resistance-conferring V1016G mutation in field populations of *Ae. albopictus* at a moderate cost. Our survey revealed the widespread distribution of the resistance allele (1016G) across Guangyuan City, albeit exclusively in heterozygous individuals (VG genotype). The absence of GG homozygotes does not necessarily reflect selection against the resistance allele, but can be attributed to (1) the relatively low frequency of the 1016G allele (3.1–14.1%), resulting in an expected homozygote prevalence below 2%, and (2) sampling limitations, where such low expected values (< 1) make observation unlikely. This interpretation is supported by the conformity of all populations to HWE (*P* > 0.05). The overall low resistance allele frequency (< 10%) and absence of GG homozygotes suggest that pyrethroids may still retain partially effective in this region. Rotational use with non-pyrethroid insecticides at this stage could help delay the fixation of resistance alleles. The lack of a significant difference in allelic frequency among populations (*P* = 0.356) implies either a similar history of insecticide use in these regions or an ongoing gene flow between populations.

Comparison with 2020 data revealed slight increases in the 1016G allele frequency in four counties/districts (Cang-Xi, Jian-Ge, Li-Zhou, and Qing-Chuan). However, these increases were not statistically significant. Overall, no significant temporal change in allele frequency was observed in any of the populations within Guangyuan City between 2020 and 2024 (*P* > 0.05; Table 3). This stability is consistent with the absence of a substantial increase in pyrethroid insecticide usage intensity in this region during the same period, as indicated through personal communication with pest control operators.

## Conclusions

We developed and validated a cost-effective and reliable PCR–RFLP assay for detecting the V1016G mutation in *Ae. albopictus*, and provided updated insights into its occurrence. This work delivers a practical tool to facilitate pyrethroid resistance surveillance and critical evidence to inform control strategies.

## Supplementary Information

Additional file 1. Fig. S1 Agarose gel electrophoresis image. (A) PCR products (~350 bp). M: DNA molecular weight marker. (B) BsaJI-digested PCR products. M: DNA molecular weight marker; Lanes 1–3: Homozygous wild-type (VV, ~350 bp); Lanes 4–7: Heterozygous (VG, ~350 bp + ~240 bp + 115 bp); Lanes 8–9: Homozygous resistant (GG, ~240 bp + 115 bp).

## Data Availability

Data supporting the main conclusions of this study are included in the manuscript.

## Abbreviations

VGSC: Voltage-gated sodium channel
PCR–RFLP: Polymerase chain reaction–restriction fragment length polymorphism
HWE: Hardy–Weinberg equilibrium
DENV: Dengue virus
AS–PCR: Allele-specific PCR
PCR–MS: PCR coupled with mass spectrometry
