# Supplementary figures and images for: Development and application of a PCR–RFLP assay revealing widespread distribution of the pyrethroid resistance-associated VGSC V1016G mutation in Aedes albopictus from Guangyuan City, Sichuan Province of China
Source: Parasit Vectors. 2025 Nov 24;18:484. doi: 10.1186/s13071-025-07116-z (PMC12642244; doi:10.1186/s13071-025-07116-z)

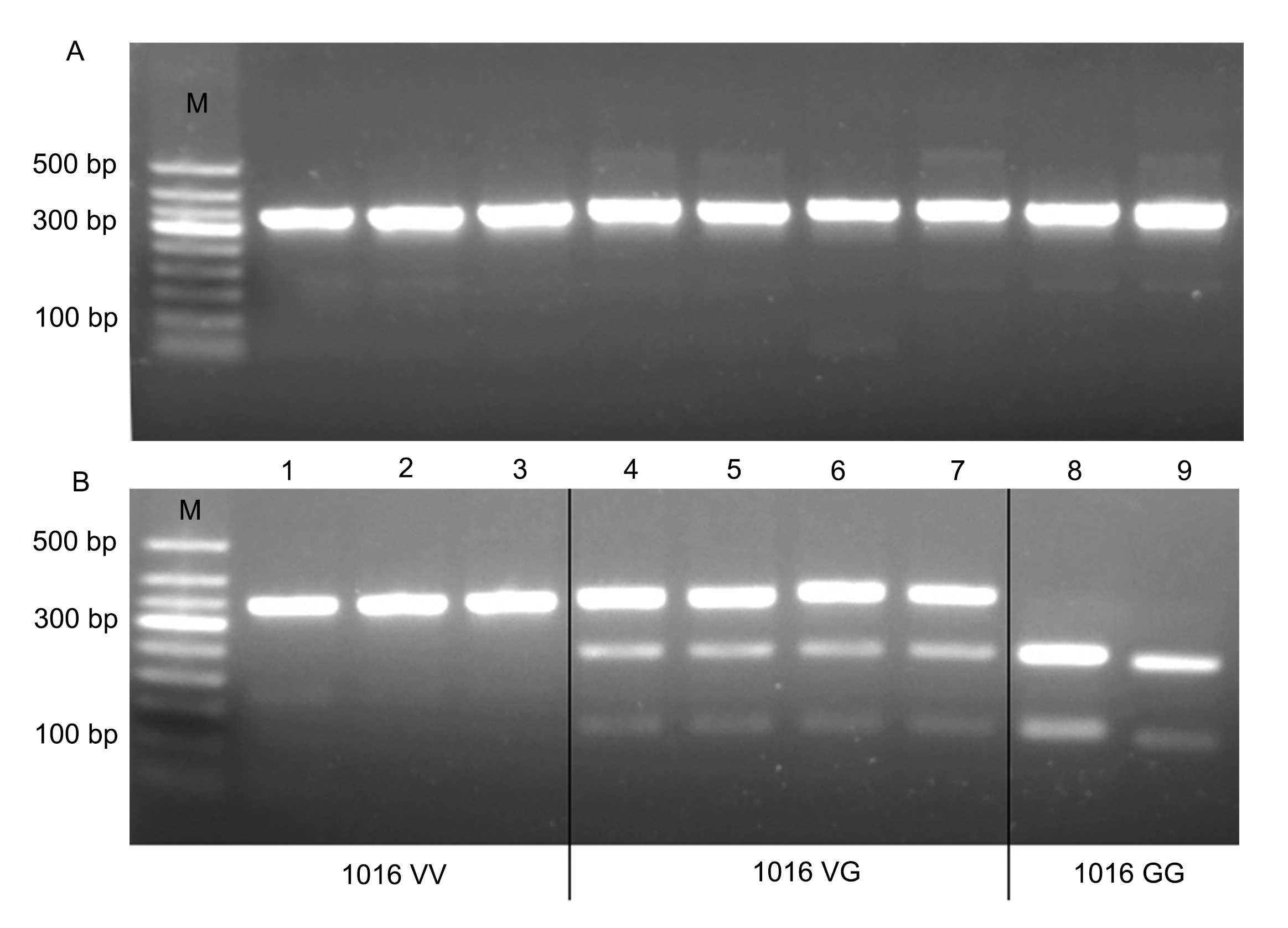

Supplement: Supplementary file 1 — Additional file 1. Fig. S1 Agarose gel electrophoresis image. (A) PCR products (~350 bp). M: DNA molecular weight marker. (B) BsaJI-digested PCR products. M: DNA molecular weight marker; Lanes 1–3: Homozygous wild-type (VV, ~350 bp); Lanes 4–7: Heterozygous (VG, ~350 bp + ~240 bp + 115 bp); Lanes 8–9: Homozygous resistant (GG, ~240 bp + 115 bp). [file 13071_2025_7116_MOESM1_ESM.jpg]
